# Angiotensin Receptor Blocker Associated with a Decreased Risk of Lung Cancer: An Updated Meta-Analysis

**DOI:** 10.3390/jpm13020243

**Published:** 2023-01-29

**Authors:** Zexu Wang, Lingyun Wei, Cheng Yin, Wang Li, Bing Wan

**Affiliations:** 1 Department of Respiratory and Critical Care Medicine, The Affiliated Jiangning Hospital of Nanjing Medical University, Nanjing 210002, China; 2 Department of Cardiothoracic Surgery, School of Medicine, Nanjing University/Jinling Hospital, Nanjing 210002, China; 3 Department of Clinical Laboratory, The Affiliated Jiangning Hospital of Nanjing Medical University, Nanjing 210002, China

**Keywords:** angiotensin receptor blockers (ARB), incidence of lung cancer, risk of lung cancer, Asian, Valsartan

## Abstract

Introduction: There have been disputes in the association between angiotensin receptor blockers (ARB) and the incidence of lung cancer. Our meta-analysis reevaluated this problem from the perspectives of race, age, drug type, comparison objects and smoking. Method: We used the following databases to carry out our literature search: Pubmed, Medline, Cochrane Library, and Ovid (From 1 January 2020 to 28 November 2021). The correlation between ARBs and the incidence rate of lung cancer was calculated by risk ratios (RRs). Confidence intervals were selected with 95% confidence intervals. Results: A total of 10 randomized controlled trials (RCTs), 18 retrospective studies and 3 case-control studies were found to satisfy the inclusion criteria. The use of ARB drugs reduced the incidence of lung cancer. The pooled results of 10 retrospective studies revealed a decreased lung cancer incidence in patients treated with ARBs, especially in patients using Valsartan. A significantly lower lung cancer incidence was found in the ARB drugs than in calcium channel blockers (CCBs) and angiotensin-converting enzyme inhibitors (ACEIs). Lung cancer occurrence was lower in Asian-based studies, especially in Mongolian-dominated and Caucasian-dominated patient populations. No significant decrease in lung cancer occurrence was found in RCTs or in patients receiving telmisartan, losartan, candesartan, irbesartan, or other placebo or in American and European-dominated patient populations. Conclusion: Compared with ACEIs and CCBs, ARBs significantly reduce the risk of lung cancer, especially in Asian and Mongolian populations. Valsartan has the best effect in reducing the risk of lung cancer in ARB drugs.

## 1. Introduction

Hypertension and cancer are the two most important fatal diseases in the world. Angiotensin receptor blockers (ARBs) are antihypertensive drugs that have complex associations with the risk of cancer and are involved in the regulation of cancer. Angiotensin II (Ang II) plays an important role in tumorigenesis by stimulating cell angiogenesis. Therefore, ARBs that block angiotensin type 2 receptors (AT2) can reduce the risk of cancer [1]. Meanwhile, ARBs may also achieve tumor growth inhibition by inhibiting lymphatic vessel growth [2] and reversing cancer-induced immunosuppression [3].

The relationship between ARBs and cancer incidence is controversial. Many meta-analyses have addressed this problem; however, small sample sizes, long publishing dates, and contradictory results limit the credibility of these studies [4,5,6,7]. Based on the analysis of randomized experiments, Sipahi et al. and the ARB Trialists Collaboration group reached different conclusions that ARBs would increase the risk of lung cancer and ARBs had no relationship with the increase in cancer incidence, respectively [6,7]. ARBs may inhibit the growth of lung cancer cells by blocking the effect of Ang II on increasing cell membrane free calcium and activating the angiotensin peptide receptor. Therefore, it is of great importance to investigate the relationship between lung cancer incidence and ARBs from the perspective of lung cancer alone. On the other hand, recently published large retrospective cohort studies may change the results.

Based on these questions, our meta-analysis focused on the relationship between lung cancer and ARB drugs, as well as the influence of race, age, drug type, comparison objects, and smoking.

## 2. Materials and Methods

### 2.1. Search Strategy

We used the following database to carry out our literature search: Pubmed, Medline, Cochrane Library, and Ovid (from 1 January 2020 to 28 November 2021). The following keywords were used: (lung cancer) AND (ARB) OR (Angiotensin Receptor Blocker).

### 2.2. Inclusive and Exclusive Criteria

The included studies adhered to the following criteria: (1) patients: patients without lung cancer before taking antihypertensive drugs; (2) interventions: the control group did not take ARB drugs, and the experimental group took ARB drugs; (3) outcome indicators: incidence of lung cancer; (4) study type: randomized controlled study (RCT) or retrospective cohort study or case-controlled studies. If the same study population was used, all articles with incomplete relative data and earlier publication time were excluded, and the remaining one was included. Studies that met the following criteria were excluded in this meta-analysis: (1) letters, reviews, editorials, comments, animal experiments, and duplicated studies; (2) studies in which data on the incidence of lung cancer were not available; and (3) manuscripts written in Chinese.

### 2.3. Quality Evaluation and Statistical Analysis 

We evaluated retrospective cohort studies and case reports using the Newcastle Ottawa scale (NOS) [8], and RCTs were evaluated using the Cochrane Risk Assessment Tool [9].

Stata 14.0 (Stata Corporation, college station, TX, USA) and Review Manager (Cochrane Collaboration, Oxford, UK) were selected to perform analyses. The relationship between ARB drugs and lung cancer incidence was calculated as risk ratios (RRs) with 95% confidence intervals (95% CI). 

Heterogeneity was evaluated using the I-squared (I^2^) test. An I^2^ > 50% was considered to indicate significant heterogeneity, and further analysis used the random-effects model. I^2^</=50% indicated acceptable heterogeneity, and a fixed-effects model was used for further analysis. If significant heterogeneity existed, sensitivity analyses were used to help determine which studies had the greatest potential impact. Heterogeneity was explained by subgroup analysis. Potential publication bias was detected using the Duval trim-and-fill method [10]. A symmetrical image indicated no publication bias. Otherwise, publication bias existed.

## 3. Results

### 3.1. Literature Selection Results and Characteristics of the Included Studies

**Figure 1:**
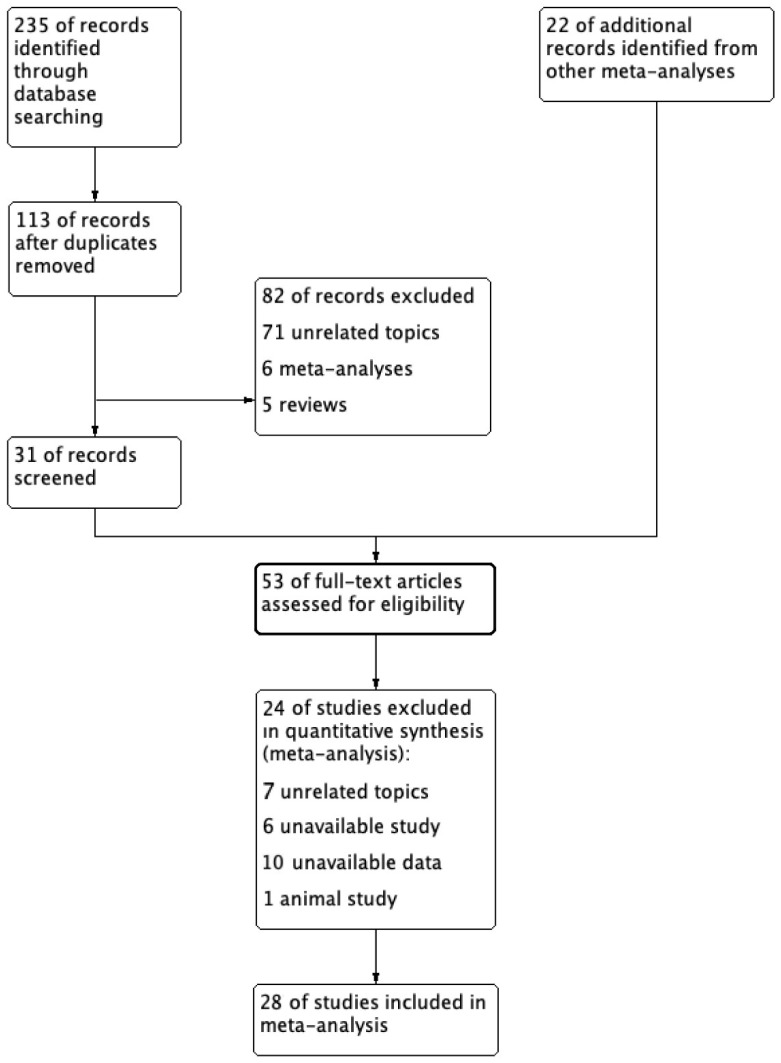
Document search process.

**Figure 2:**
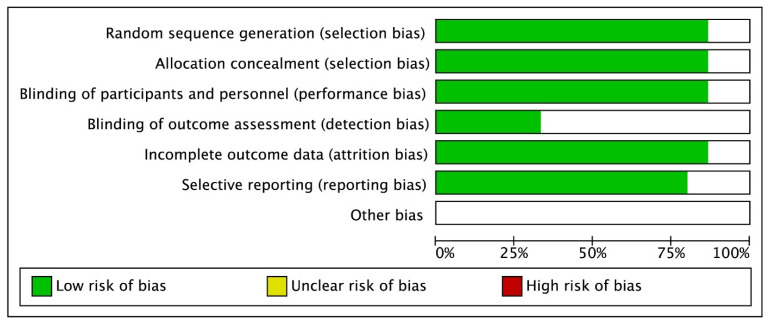
Quality Evaluation Results of RCTs.

A total of 235 studies selected from database searching and 22 studies from other meta-analyses were included in the initial screening. Two reviewers screened all studies independently (Figure 1). After screening duplicated literature and reviewing abstracts and full texts, 28 studies were included in this meta-analysis. There were 10 retrospective cohort studies, 15 RCTs, and 3 case-controlled studies [11,12,13,14,15,16,17,18,19,20,21,22,23,24,25,26,27,28,29,30,31,32,33,34,35,36,37,38,39]. Among them, Jung et al. [11] included two cohort study populations, which were included. The characteristics of the studies are summarized in Table 1. Retrospective studies were evaluated using the NOS (Table 1). Bias assessment for the ten RCTs was performed using the Cochrane collaboration tool (Figure 2).

### 3.2. Overall Analysis 

**Figure 3:**
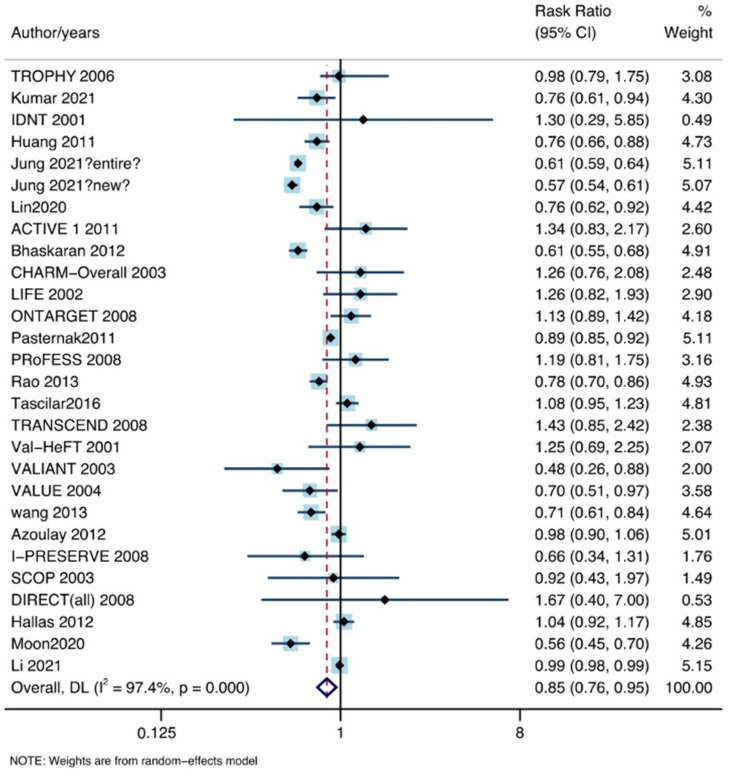
The association between ARB drugs and incidence of lung cancer [11,12,13,14,15,16,17,18,19,20,21,22,23,24,25,26,27,28,29,30,31,32,33,34,35,36,37,38,39].

In total, 28 studies, including 6,301,712 patients, satisfied the inclusion criteria. The use of ARB drugs reduced the incidence of lung cancer (RR: 0.85, 95% CI lower: 0.76, 95% CI upper: 0.95) under the random effects model (I^2^ = 97.4%, P = 0.00; Figure 3).

**Figure 4:**
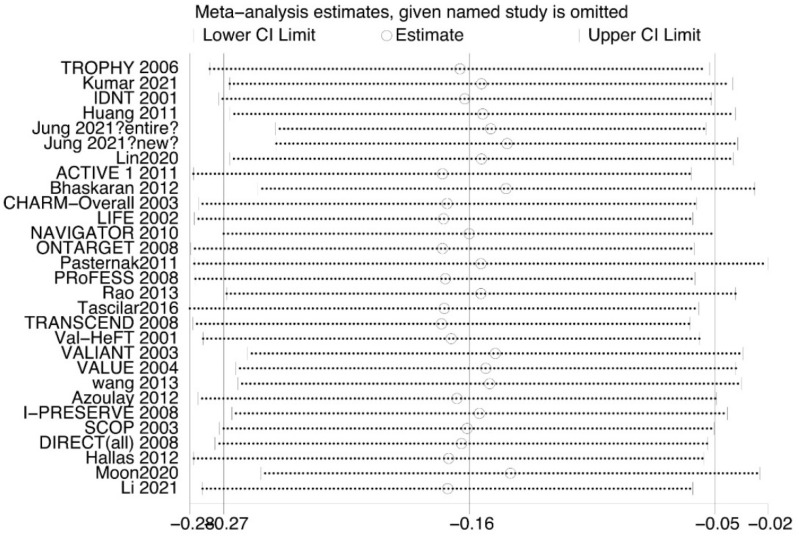
Sensitivity analysis of all studies [11,12,13,14,15,16,17,18,19,20,21,22,23,24,25,26,27,28,29,30,31,32,33,34,35,36,37,38,39].

**Figure 5:**
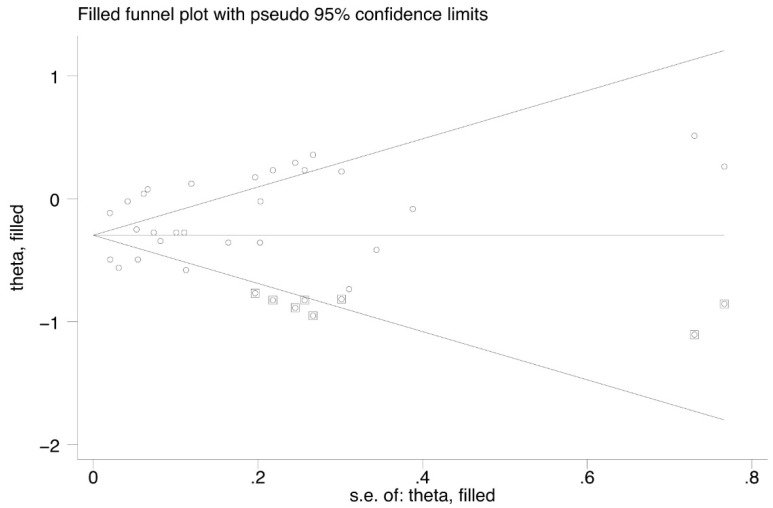
Funnel plot of nonparametric trim-and-fill method.

Our sensitivity analysis (Figure 4) showed that Jung et al. and Pasternak et al. significantly affect the heterogeneity. The nonparametric trim-and-fill method suggested the existence of publication bias (Figure 5).

### 3.3. Subgroup Analysis by Study Type

**Figure 6:**
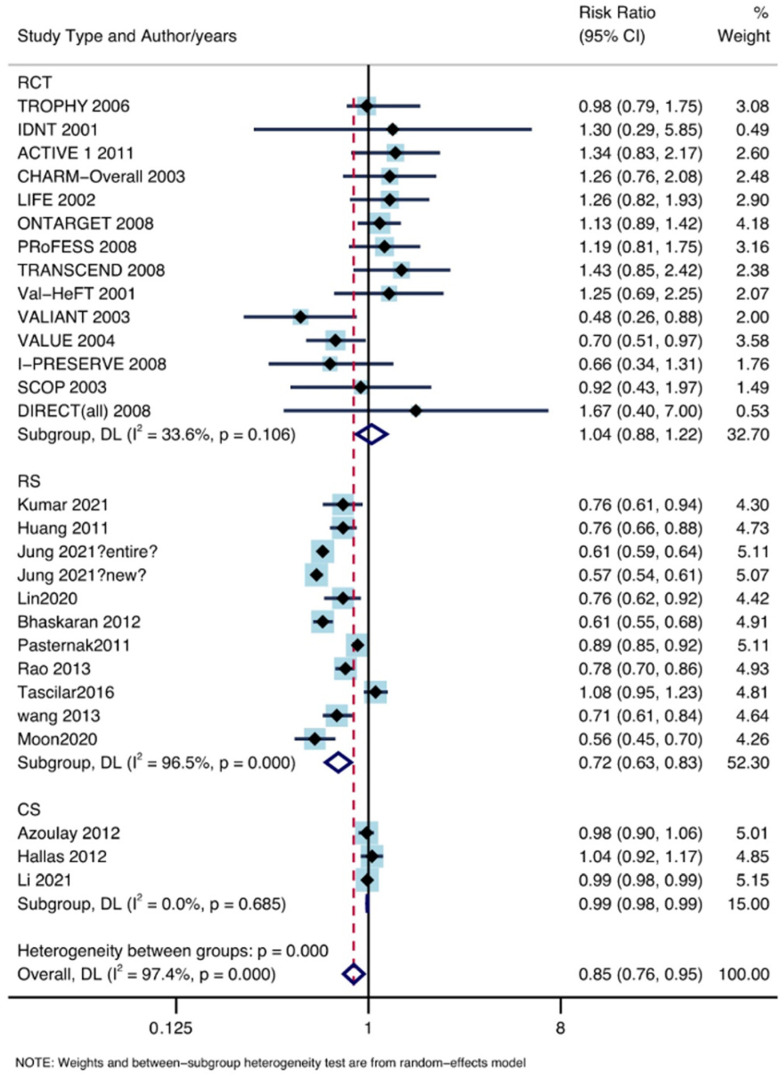
Subgroup analysis by study type [11,12,13,14,15,16,17,18,19,20,21,22,23,24,25,26,27,28,29,30,31,32,33,34,35,36,37,38,39].

As shown in Figure 6, 10 retrospective studies, including 5,453,716 patients; 14 randomized-controlled studies, including 126,005 patients; and 3 case reports, including 712,798 patients, reported the lung cancer incidence rate in patients using ARB drugs. The pooled results of retrospective studies revealed a reduced incidence of lung cancer in patients using ARB drugs (RR: 0.72, 95% CI lower: 0.63, 95% CI upper: 0.83). Significant heterogeneity was found (I^2^ = 96.5%, P = 0.00). A decreased incidence of lung cancer was found in CSs (RR: 0.99, 95% CI lower: 0.98, 95% CI upper: 0.99; I^2^ = 0%, P = 0.685). No significantly decreased incidence of lung cancer was found in RCTs (RR: 1.04, 95% CI lower: 0.88, 95% CI upper: 1.22; I^2^ = 33.6%, P = 0.106).

### 3.4. Subgroup Analysis According to ARB Drugs

**Figure 7:**
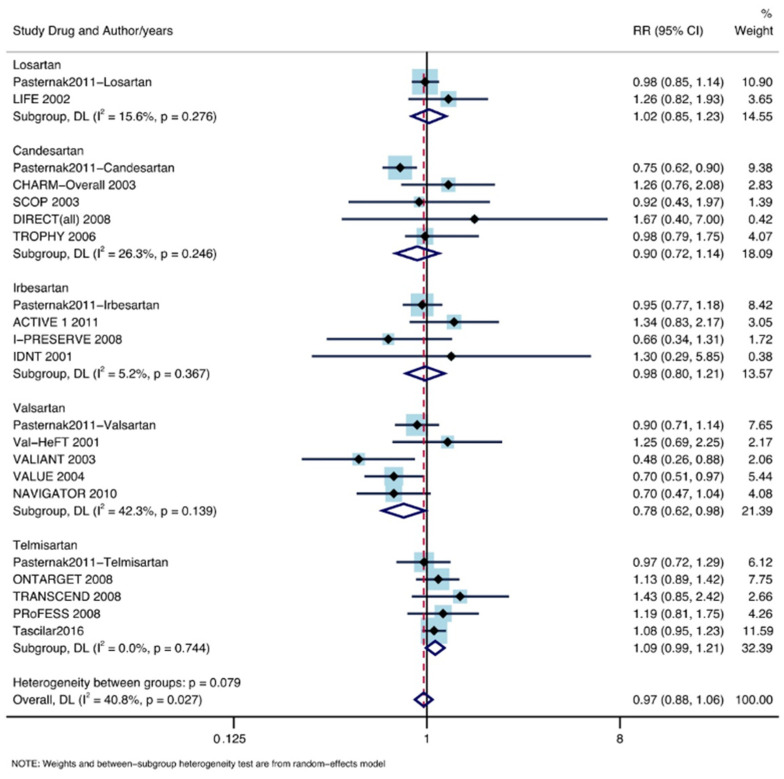
Subgroup analysis by different ARB drugs [11,12,13,14,15,16,17,18,19,20,21,22,23,24,25,26,27,28,29,30,31,32,33,34,35,36,37,38,39].

Of the patients included, 44,025 took Valsartan, 111,799 took telmisartan, 9193 took losartan, 18,008 took candesartan, and 14,284 took irbesartan. A significantly decreased lung cancer occurrence was found in patients treated with Valsartan (RR: 0.78, 95% CI lower: 0.62, 95% CI upper: 0.98; I^2^ = 42.3%, P = 0.139). No significant decline in the incidence of lung cancer was found in patients taking telmisartan (RR: 1.09, 95% CI lower: 0.99, 95% CI upper: 1.21; I^2^ = 0%, P = 0.744), losartan (RR: 1.02, 95% CI lower: 0.85, 95% CI upper: 1.23; I^2^ = 15.6%, P = 0.276), candesartan (RR: 0.90, 95% CI lower: 0.72, 95% CI upper: 1.14; I^2^ = 26.3%, P = 0.246), or irbesartan (RR: 0.98, 95% CI lower: 0.80, 95% CI upper: 1.21; I^2^ = 5.2%, P = 0.367). The forest plot is shown in Figure 7.

### 3.5. Subgroup Analysis by Comparison Object

**Figure 8:**
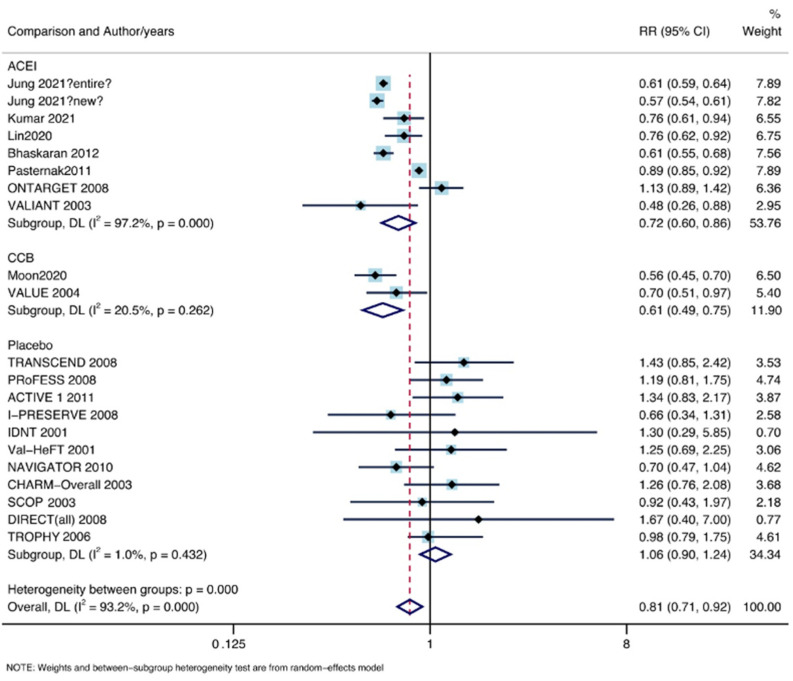
Subgroup analysis by comparison [11,12,13,14,15,16,17,18,19,20,21,22,23,24,25,26,27,28,29,30,31,32,33,34,35,36,37,38,39].

As shown in Figure 8, eight control groups, including 3,946,473 patients, received ACEIs; two control groups, including 74,207 patients, received CCBs; and 11 control groups of 72,187 patients received other types of placebo. A significant decrease in lung cancer was found in the ARBs compared with the ACEIs (RR: 0.72, 95% CI lower: 0.60, 95% CI upper: 0.86; I^2^ = 97.2%, P = 0.00) and CCBs (RR: 0.61, 95% CI lower: 0.49, 95% CI upper: 0.75; I^2^ = 20.5%, P = 0.262). No significantly decreased incidence of lung cancer was found in the other placebo group (RR: 1.06, 95% CI lower: 0.90, 95% CI upper: 1.24, I^2^ = 1%, P = 0.00).

### 3.6. Subgroup Analysis by Continent

**Figure 9:**
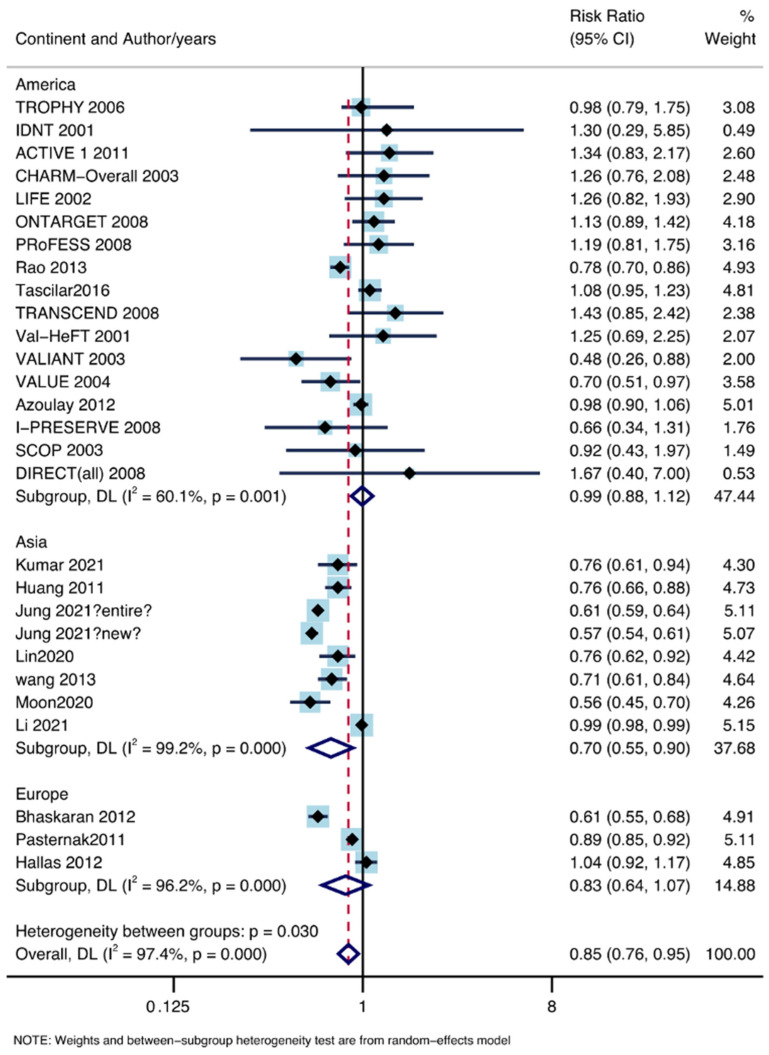
Subgroup analysis by continent [11,12,13,14,15,16,17,18,19,20,21,22,23,24,25,26,27,28,29,30,31,32,33,34,35,36,37,38,39].

Eight Asian-based studies with 3,647,607 patients, 18 American-based studies with 1,878,434 patients, and three European-based studies with 775,671 patients were included. The RR value of Asian-based studies was 0.70, and the 95% CI was 0.55–0.90, which indicated decreased lung cancer incidence rate under the use of ARB drugs (I^2^ = 99.2%, P = 0.00). No meaningful result was obtained in the summary analysis of American-based studies (RR: 0.97, 95% CI lower: 0.87, 95% CI upper: 1.09; I^2^ = 59.8%, P = 0.001) and European-based studies (RR: 0.83, 95% CI lower: 0.64, 95% CI upper: 1.07; I^2^ = 96.2%, P = 0.00). The forest plot is shown in Figure 9.

### 3.7. Subgroup Analysis by Main Race

**Figure 10:**
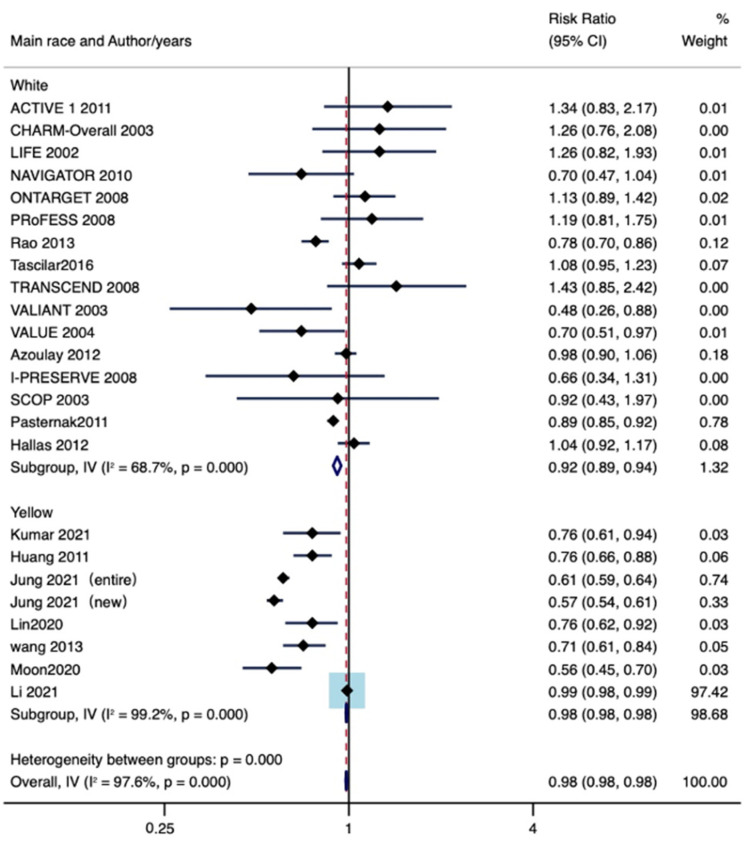
Subgroup analysis by main race [11,12,13,14,15,16,17,18,19,20,21,22,23,24,25,26,27,28,29,30,31,32,33,34,35,36,37,38,39].

The main race was defined as the race that accounts for more than half of the total study population. A total of 19 studies, including 2,648,100 patients, were mainly Caucasian, and seven studies, including 3,647,607 patients, were mainly Mongolian. As shown in Figure 10, lung cancer incidence decreased in Mongolian-dominated patient populations (RR: 0.98, 95% CI lower: 0.98, 95% CI upper: 0.98 I^2^ = 99.2%, P = 0.00) and in Caucasian-dominated patient populations (RR: 0.92, 95% CI lower: 0.89, 95% CI upper: 0.94; I^2^ = 68.7%, P = 0.00).

## 4. Discussion

This meta-analysis examined a number of studies to explore the relationship between lung cancer incidence rate and ARB drug usage and compared different ARB drugs and other antihypertensive drugs in a categorical manner to explore this issue in a comprehensive way. We also conducted separate analyses according to population characteristics to explore the impact of patient characteristics on the association between drugs and disease.

To attempt to resolve the previous controversy [4,5,6,7], we included seven new large retrospective cohort studies on the basis of the original experimental studies. We found that ARB drugs are linked to a decreased incidence of lung cancer. Unlike other meta-analyses, this is the first to indicate that Valsartan significantly reduces the incidence of lung cancer in comparison with ACEIs and CCBs. A benefit in terms of reduced incidence of lung cancer was detected in Asians and Mongolians using ARB drugs for the first time. 

This is the first study to link a single category of ARB drugs with the incidence of lung cancer and discuss the relationship between them. Although no significant difference was observed in all ARB drugs, the conclusion that Valsartan can reduce lung cancer risk by 22% is considerable. Sacubitril/Valsartan is an antihypertensive drug whose efficacy has been demonstrated in recent years. In comparison with enalapril, an angiotensin-converting enzyme (ACE) inhibitor, it has a better effect in reducing the incidence rate and mortality rate of heart failure patients with decreased ejection fraction [40,41,42]. However, few scholars have focused on the relationship between Valsartan and cancer, and additional clinical evidence is needed.

Compared to ACEIs and CCBs, ARB drugs were related to the reduction of lung cancer incidence. This is inconsistent with the results of the ARB trialist collaboration, which reported no significant difference between ACEIs and ARBs [7]. Unlike the statistical method of the ARB collaboration, the present ACEI control group only used ACEIs and excluded patients who used other drugs that may affect blood pressure at the same time. Therefore, the present results exclude the interference of other antihypertensive drugs and focus on the different effects of two antihypertensive drugs on lung cancer risk. In addition to the 15 RCTs included by the ARB collaboration, the present study included retrospective cohort studies published in recent years, which enriches the composition of our included articles and makes the results more reliable.

Our study indicates that the use of ARBs significantly reduces lung cancer occurrence in Asians. Similar results were reported in a meta-analysis published by Zhang et al. based on an Asian population of 298,000 individuals [43]. This study included a greater number of RCTs and retrospective cohort studies, with a total number of included people of 6 million, to make this result more convincing.

Many experiments have reported possible molecular links between ARBs and cancer. ARBs inhibit tumor cell growth by blocking signaling pathways downstream of angiotensin II Type 1 receptor (AT1R) [44,45,46], activating peroxisome proliferator-activated receptors(PPARs) [47,48,49], and inducing G0/G1 cell cycle arrest [50,51]. On the other hand, ARBs exert anti-tumor effects by increasing cancer cell autophagy through the induction of autophagy-related cell death and anti-metastatic activity [52], downregulating Bcl-2 and engaging in caspase-3-induced apoptosis pathways in cancer cells [53]. Of particular note, in lung adenocarcinoma cells, candesartan enhanced their susceptibility to tumor necrosis factor-related apoptosis-inducing ligand (TRAIL)-mediated apoptosis [54]. However, the potential risk of ARBs on tumors has also been reported in certain studies. Telmisartan was found to promote tumor cell growth by improving tumor cell microcirculation [55], and cloxacin significantly increased cell adhesion and invasion on a type I collagen matrix [56]. Thus, our study demonstrates a favorable association between ARBs and lung cancer, and the molecular basis of this association remains to be investigated.

The nonparametric trim-and-fill method showed the existence of publication bias, and sensitivity analysis revealed that experiments by Jung et al. and Pasternak et al. may have caused significant heterogeneity. We reduced this bias by performing subgroup analysis to improve the reliability of the results. The present study had several limitations. The heterogeneity was high among the studies. Our sensitivity analysis showed that this could be attributed to studies by Jung et al. and Pasternak et al. However, we did not have access to raw data, which may result in data deviation. Finally, without the raw data, it was difficult to examine this issue at a comprehensive level.

## 5. Conclusions

The present meta-analysis indicates that, compared to ACEIs and CCBs, ARB drugs can significantly reduce the incidence of lung cancer. Among the ARBs analyzed, Valsartan was the most effective drug for reducing lung cancer incidence. ARB drugs can reduce the risk of lung cancer in Asian, Mongolian and Caucasian hypertensive patient populations.

## Figures and Tables

**Table 1:** The characteristics of the included studies.

Author/Years	Study Type	Main Race	Continent	Mean Age	Study Drug	Comparison	QE
Jung 2021 (entire) [11]	RS	Yellow	Asia	56.9	NA	ACEI	6
Jung 2021 (new) [11]	RS	Yellow	Asia	56.5	NA	ACEI	6
Kumar 2021 [12]	RS	Yellow	Asia	49.6	NA	ACEI	5
Lin2020 [13]	RS	Yellow	Asia	58.9	NA	ACEI	8
Moon2020 [14]	RS	Yellow	Asia	NA	NA	CCB	8
Bhaskaran 2012 [15]	RS	White	Europe	64	NA	ACEI	8
Huang 2011 [16]	RS	Yellow	Asia	58.5	NA	non-ARB	5
Rao 2013 [17]	RS	White	America	63	NA	non-ARB	8
wang 2013 [18]	RS	Yellow	Asia	62	NA	non-ARB	7
Tascilar2016 [19]	RS	White	America	62.5	Telmisartan	Other ARBs	7
Pasternak2011 [20]	RS	White	Europe	64.3	NA	ACEI	8
ONTARGET 2008 [21]	RCT	White	America	66.4	Telmisartan	Ramipril	-
TRANSCEND 2008 [22]	RCT	White	America	66.9	Telmisartan	Placebo	-
PRoFESS 2008 [23]	RCT	White	America	66.1	Telmisartan	Placebo	-
ACTIVE 1 2011 [24]	RCT	White	America	69.5	Irbesartan	Placebo	-
I-PRESERVE 2008 [25]	RCT	White	America	72	Irbesartan	Placebo	-
IDNT 2001 [26]	RCT	White	America	59.3	Irbesartan	Placebo	-
Val-HeFT 2001 [27]	RCT	White	America	62.7	Valsartan	Placebo	-
VALIANT 2003 [28]	RCT	White	America	64.8	Valsartan	captopril	-
VALUE 2004 [29]	RCT	White	America	67.3	Valsartan	Amlodipine	-
NAVIGATOR 2010 [30]	RCT	White	America	63.7	Valsartan	Placebo	-
CHARM-Overall 2003 [31]	RCT	White	America	65.9	Candesartan	Placebo	-
TROPHY 2006 [32]	RCT	NA	America	48.5	Candesartan	Placebo	-
DIRECT (all) 2008 [33,34]	RCT	NA	America	NA	Candesartan	Placebo	-
SCOP 2003 [35]	RCT	White	America	76.4	Candesartan	Placebo	-
LIFE 2002 [36]	RCT	White	America	66.9	Losartan	Atenolol	-
Hallas 2012 [37]	CS	White	Europe	NA	NA	non-ARB	6
Azoulay 2012 [38]	CS	White	America	72.4	NA	non-ARB	8
Li 2021 [39]	CS	Yellow	Asia	NA	NA	Non-ARB	8

Abbreviation: RS: retrospective study; RCT: randomized-controlled study; CS: case report. QE: quality evaluation. NA: not acquired.

## Data Availability

No new data were created or analyzed in this study. Data sharing is not applicable to this article.
